# Editorial: Climate adaptations and challenges of non-native tree species in forest ecosystems

**DOI:** 10.3389/fpls.2026.1768503

**Published:** 2026-02-24

**Authors:** Marcin Klisz, Giovanna Battipaglia, Mathieu Lévesque, Sergio Rossi

**Affiliations:** 1 Dendrolab IBL, Department of Silviculture and Genetics, Forest Research Institute, Sekocin Stary, Poland; 2 Department Environmental, Biological and Pharmaceutical Sciences and Technologies, University of Campania Luigi Vanvitelli, Sekocin Stary, Italy; 3 Silviculture Group, Institute of Terrestrial Ecosystems, ETH Zurich, Universitätstrasse, Zurich, Switzerland; 4 Département de Sciences Fondamentales, Université du Québec à Chicoutimi, Chicoutimi, QC, Canada

**Keywords:** acclimatisation, climate change, growth, introduced tree species, woody species

Non-native tree species (NNTs) are an important component of the forest resource worldwide, although their distribution across climatic zones remains heterogeneous (Speziale et al., 2012; Brus et al., 2019). As declines in forest condition become increasingly evident at the global scale, the ecosystem functions related to productivity, ecology and society provided by NNTs are increasingly at risk (Castro-Diez et al., 2019). In turn, some of these species considerably affect ecological conditions, contributing to a general decline in the local biodiversity (Wohlgemuth et al., 2022). Understanding the adaptation of NNTs to climate change and their impacts on ecosystem services is therefore of growing interest in the context of biological conservation, forest ecology and management (Novoa et al., 2024). The history of the introduced species and their acclimatisation are particularly important, not only for their potential as alternatives to native species (Thurm et al., 2018), but also for their possible high invasiveness and the threat they may pose to the biodiversity of native habitats, often in competition with local species (Brundu et al., 2020).

Despite numerous studies on the acclimatisation of NNTs, their interaction with native tree species or, more generally, their influence on the functioning of host habitats, our knowledge is still far from complete. Research projects and other scientific initiatives focus on selected NNTs, ignoring others that are less common in the place of their introduction (Dimitrova et al., 2022) or whose importance for forestry is marginal (Schlaepfer et al., 2020). The uneven distribution of scientific efforts, mainly focus on Europe and North America, results in knowledge about NNTs in other regions being fragmentary or even non-existent (Castro-Diez et al., 2019). While production potential, invasiveness and ecosystem service provision are extensively addressed in numerous studies, many other Research Topics still require attention from scientists.

The process of acclimatisation of NNTs and their potential benefits and disadvantages in forestry require a close assessment of their growth and reproductive behaviour under the new environmental conditions (Bouteiller et al., 2021; Klisz et al., 2025). In particular, there is a need for retrospective approaches to quantify the physiological processes of NNTs, which can be achieved through long-term monitoring on permanent plots (Hoffmann et al., 2020; Gręda et al., 2022). Common garden experiments are especially valuable, as they enable the disentanglement of inter- and intraspecific variation in phenotypic traits (Alizoti et al., 2022). These approaches should be complemented by experiments conducted under controlled conditions that simulate the climatic regimes projected for the coming decades (Medina et al., 2024). Intraspecific variation in adaptive potential, especially in species with a wide ecological range, requires consideration of populations from both the edge and the core of the distribution.

This Research Topic includes studies addressing the acclimatisation and the adaptive potential of non-native woody species to changing climatic conditions, covering a wide range of climate zones, various taxonomic groups, plant developmental phases, and physiological processes. This collection of scientific papers aims to encourage scientists from various disciplines to present innovative, cutting-edge research on the broadly understood climate change adaptation of NNTs. In response to the call for papers, we published nine high-quality submissions from 35 leading researchers representing nine countries across three continents (**Table 1**). We are pleased to present a collection of high-quality publications on the Research Topic.

**Table 1:** Summary of studies included in Research Topic.

Study	Species	Site/country	Main goal	Methods
Ali et al.	*P. juliflora* *P. cineraria*	Muscat (Oman)	Test how soil conditioning with native and invasive species will affect plant performance and production.	Two-phase experiment with soil conditioning phase and feedback phase.
Camarero et al.	*L. decidua*	Valgañón, Santurde, Ribavellosa, Santa Marina, (Spain)	Test tolerance to climate and drought stress	Dendroclimatic analysis
Griesbauer et al. (2025)	*P. menziesii* *L. occidentalis* *P. ponderosa*	British Columbia (Canada)	Examine variation in tree productivity across a range of environments located within and outside their current natural geographic range.	Dendroecological analysis
Moser et al.	*P. menziesii* *P. abies* *A. alba* *P. sylvestris* *F. sylvatica* *Q. robur* *Q. petraea* *A. pseudoplatanus*	Birmensdorf (Switzerland)	Test performance of *P. menziesii* seedlings growing in competition with native conifer and broadleaf trees.	Common garden experiment
Spangenberg et al.	*P. menziesii*	Baden-Württemberg (Germany)	Test the effect of drought stress on *P. menziesii* growth during 2018 drought.	Stem size growth monitoring.
Niessner et al.	*P. menziesii*	Brandenburg (Germany)	Test the effect of soil drying on *P. menziesii* growth during 2022 summer drought.	Stem size growth and sap flow monitoring.
Kormann et al.	*Q. rubra*	Dunkelsdorf, Waldsieversdorf, Waldsieversdorf (Germany)	Test provenance-specific climate sensitivity of *Q. rubra.*	Dendroecological analysis
Wang and Dang	*A. ginnala* *A. negundo*	Ontario (Canada)	Test CO_2_ effect on photosynthetic efficiency and growth.	Greenhouse experiment with CO_2_ and N manipulation.
Konic et al.	*P. menziesii* *A. grandis* *T. plicata* *P. radiata* *P. contorta* *R. pseudoacacia* *Q. rubra* *F. nigra*	Austria	Assess the role of NNTs in facilitating forest adaptation to climate change to secure ecosystem services.	Species Distribution Models

## 1 Invasive species and colonised habitat interactions

Introduced tree species with high invasive potential develop complex interactions with the components of the host environment, whether the atmosphere, soil, or biotope. These plants can strongly modify soil conditions, while global warming, atmospheric nitrogen deposition, and elevated CO_2_ concentration can modify their photosynthetic efficiency or increase nitrogen concentration in plant tissue. As part of this compilation, Ali et al. found that *Prosopis juliflora* (Sw.) DC., an invasive species in the Middle East, significantly alters soil properties significantly, mainly affecting soil organic carbon and soil total nitrogen. This leads to positive plant-soil feedback (PSF) in this species, whereas negative PSF was observed in the native *Prosopis cineraria* (L.) Druce. This undesired interaction between an alien and invasive species and soil conditions raises concerns about whether the restoration of native *P. cineraria* is at risk. The strategy of adaptation to climate change, expressed in high efficiency of resource acquisition, photosynthesis rate, and consequently faster investment returns, may indicate high plasticity of invasive species. The increase in atmospheric CO_2_ concentration, predicted in many climate change scenarios, is likely to promote higher photosynthetic efficiency, stomatal conductance, and, as a result, increased biomass production in two invasive maple species in Europe, *Acer ginnala* (Maxim.) Wesm. and *Acer negundo* L. On the other hand, increased nitrate deposition observed in many regions, unlike ammonium fertilisation, may significantly limit photosynthetic efficiency and thus reduce the invasive potential of these two introduced maple species Wang and Dang.

## 2 Outside geographical range, yet within fundamental niches

Assisted colonisation of tree species outside their natural range often aims to introduce species into areas that lie within their fundamental niches but are impossible to colonise due to geographical or anthropogenic barriers (e.g., deforested areas) (Gardner and Bullock, 2025). In such situations, it is assumed that the adaptive plasticity of species will enable them to acclimatise to new conditions. Performance under new conditions depends on the adaptive strategies adopted by NNTs. In this Research Topic, Camarero et al. found that it result in a survival-at-any-cost approach under stressful conditions, but may also lead to competing strategy with local species when resources are unlimited. With adverse growth conditions arising from severe drought episodes, *Larix decidua* Mill. prioritises hydraulic safety over hydraulic efficiency. This strategy, however, leads to reduced growth and, consequently, reduced competitiveness with native species. Although this strategy may facilitate tree establishment and persistence under current climatic conditions, it does not ensure long-term success as environmental conditions become increasingly unfavourable and potentially exceed species-specific ecological tolerance limits. However, assisted range expansion may lead to increased productivity and even effective competition with local species. (Griesbauer et al., 2025) suggested in this Research Topic that certain species may still have unrealised niche space, which should be addressed when projecting their potential ranges under a changing climate.

## 3 Coping with adverse conditions – insights from different ontogenetic stages

The increasing sensitivity to climate change observed in Central European conifers has triggered interest in NNTs as potential alternatives. *Pseudotsuga menziesii* (Mirbel) Franco, the most common NNT in Central Europe due to its drought tolerance, is now in focus. Resistance to adverse growing conditions applies to all stages of ontogenetic development; therefore, early-life history traits under differing resource availability conditions provide key information about the potential of seedlings to cope with unfavourable conditions and compete with native species. In this Research Topic, Moser et al. pointed out that under reduced water availability and limited nutrients, seedlings of *P. menziesii* are able to establish effectively and grow vigorously due to their high phenotypic plasticity, thus successfully competing with native conifers *P. abies*, *P. sylvestris*, and *A. alba* Mill. In its mature phase, the deep and dense root system of *P. menziesii* plays a key role in its ability to endure drought. However, Spangenberg et al. note in the same compilation that these species-specific abilities determined by the root system may be limited by soil texture. Therefore, *P. menziesii* may be vulnerable to soil water deficit under prolonged drought and deeper soil desiccation. On the other hand, Niessner et al. point out that *P. menziesii*’s specific ability to cope with drought gives it an advantage over *P. abies*. Under soil drought, this species is forced to reduce effective water transport in the trunk and xylem production to avoid cavitation and xylem dysfunction at critical xylem water potential. This tendency follows the soil moisture gradient, indicating that *P. menziesii* has already acclimatised to local conditions in Europe, as evidenced for other coniferous NNTs (Klisz et al., 2023).

## 4 Intraspecific variation – acclimatised versus native populations

Assisted acclimatisation of North American tree species utilised diverse seed sources, reflecting the natural genetic variability of native populations and thus increasing the chances of successful acclimatisation to new conditions (Neophytou et al., 2020). A reliable assessment of provenance-specific variation in the adaptation of NNTs requires unified growing conditions in which populations are tested, i.e., common-garden experiments. Such experiments are remarkably rare for species introduced in Europe, and therefore constitute an exceptionally valuable source of knowledge not only about intraspecific diversity but also about their adaptive potential to climate change (Alizoti et al., 2022). Due to the long history of introducing tree species to Europe (Bucharova and Van Kleunen, 2009), European populations of NNTs have evolved under new climatic and ecological conditions over centuries, adapting to new growing conditions. Common gardens with NNTs were established to assess intraspecific variation in growth performance under local climatic conditions, yet among factors impeding tree species performance and growth are climate change-driven anomalies (e.g., severe droughts, late frosts), which are becoming more frequent (Vitasse et al., 2019). Therefore, assessing provenance-specific growth performance in the context of their drought and frost hardiness provides key insights into their adaptive potential. Writing for this Research Topic Kormann et al. compared native and introduced populations of *Quercus rubra* L. and found that populations acclimatised to central European conditions were better adapted to extreme droughts and late frosts (namely, they have greater growth and resistance to droughts and frosts) than native populations from North America.

## 5 NNTs - solution to the decline in ecosystem service provision?

Ongoing climate change is disrupting the provision of ecosystem services by native tree species; therefore, the role of NNTs as alternative tree species is becoming particularly important. However, it is not yet certain whether promoting NNTs in forests will reverse the decline in the provision of ecosystem services. In this Research Topic Konic et al. highlighted that mixed stands, with native and introduced species, can improve productivity and tree species richness under Central European conditions; however, they are not an adequate solution for maintaining a sufficient level of protection against gravitation hazards in mountainous areas. Therefore, planning the use of NNTs should be tailored to the region-specific ecosystem service needs they are intended to support.

## 6 Future prospects and research needs

Climate change will likely shift the climatic niches of NNTs, thereby causing a contraction of their climatic optima or an expansion beyond their secondary ranges, with unknown consequences (Nicolescu et al., 2026). Therefore, it is essential to determine the direction and rate of these changes under projected climate change scenarios (Puchałka et al., 2023). However, research on the ecological niche dynamics of NNTs requires complete and up-to-date data on their present distribution. The most effective approach to obtaining up-to-date distribution data appears to be combining multiple distribution data sources, such as biodiversity, forest inventory and citizen science databases (Heberling and Isaac, 2018; Jaric et al., 2020). As data on NNTs distribution become more complete, the stability of models and the reliability of forecasts will improve. Including additional ecological niche parameters, such as soil properties and stand parameters, alongside high-resolution climate data would enhance the robustness of the models (Thuiller et al., 2019). Furthermore, most current species distribution models (SDMs) for NNTs ignore the ontogenetic development of trees, so addressing both adults (reproducing trees) and juveniles (natural regeneration) would allow verification of the overlap between reproduction and regeneration niches, hence increasing the robustness of SDMs.
